# Systematic review of hormonal strategies to improve fertility in rams

**DOI:** 10.1590/1984-3143-AR2024-0007

**Published:** 2024-06-10

**Authors:** Estela Garza-Brenner, Fernando Sánchez-Dávila, Keyla Mauleón-Tolentino, Cecilia Carmela Zapata-Campos, Carlos Luna-Palomera, Javier Hernandez-Melendez, Marisol Gonzalez-Delgado, José Fernando Vázquez-Armijo

**Affiliations:** 1 Facultad de Agronomía, Posgrado Conjunto, Universidad Autónoma de Nuevo León, General Escobedo, N.L México; 2 Facultad de Medicina Veterinaria y Zootecnia, Universidad Autónoma de Tamaulipas, Ciudad Victoria, Tamaulipas, México; 3 División Académica de Ciencias Agropecuarias, Universidad Juárez Autónoma de Tabasco, Villahermosa, Tabasco, México; 4 Facultad de Ingeniería y Ciencias, Universidad Autónoma de Tamaulipas, Tamaulipas, México; 5 Centro de Investigación en Producción Agropecuaria, Universidad Autónoma de Nuevo León, Linares, Nuevo León, México; 6 Centro Universitario Temascaltepec, Universidad Autónoma del Estado de México, Temascaltepec, México

**Keywords:** rams, kisspeptin, eCG, PGF2α, GnRH

## Abstract

Reviewing the current state of knowledge on reproductive performance and productive traits in rams has many advantages. First, the compilation of this information will serve as a literature resource for scientists conducting research around the world and will contribute to the understanding of the data collected and interpreted by researchers on the different hormonal strategies used to improve reproductive performance in rams. Second, it will allow scientists to identify current knowledge gaps and set future research priorities in ram reproduction. Rams play an important role in the global flock economy, but their reproductive analysis has been limited in the use of hormonal technologies to increase the productivity of sheep flocks. In this review, we cite the most important works on six hormones that, in one way or another, modify the hypothalamus-pituitary-gonadal axis, at different doses, in and out of the reproductive season, breeds, application methods, among other factors. The overall aim is to increase the reproductive efficiency of rams in different scenarios and, in some cases, of other species due to the lack of limited information on rams.

## Introduction

According to the FAO, by 2023 (FAO, 2023), the global sheep population will be 1,195 million head, with 42% concentrated in Asia, followed by 24% in Africa. Only 8.1% of the world's sheep are concentrated in the Americas (FAO, 2023). Sheep are the major contributors to global livestock production in virtually all agro-ecological regions (Macías-Cruz et al., 2018a, b). In many parts of the world, sheep are produced extensively in arid, semi-arid, tropical and subtropical regions with little or no nutrient supplementation (Sánchez-Dávila et al., 2020; Stewart et al., 2021). Under grazing conditions, or in less favourable environments, nutrition plays a determining role in the reproductive process of the ram, but the photoperiod is one of the main factors that determine the seasonality of the ram (Rosa y Bryant, 2003; Gündoğan et al., 2003). Hormonal concentrations of LH, FSH, testosterone and prolactin show seasonal variations, which are mainly regulated mainly by photoperiod. Therefore, a decrease in photoperiod stimulates the secretion of LH and FSH by the pituitary gland, resulting in increased testicular activity in testosterone production (Cruz-Espinoza et al., 2021).

Genetic variation in the reproductive potential among populations is large, and genetic variation within populations is often sufficient for reproductive genetic improvement (Sánchez-Dávila et al., 2011; Pérez et al., 2020). This reproductive potential can be strongly channelled to take advantage of the long reproductive season, ovulation rate and age at first lambing that these breeds present, and linking them to good management, feeding and natural resources of each region where they are used globally (Sánchez-Dávila et al., 2011.

It is mentioned that at the time of mating, because of seasonality, about 75% of the rams are optimal for breeding (Pool et al., 2020a), so the implementation of a breeding management program, with the same attention that is paid to the ewe, should be applied to the males that will be used for breeding. In this context, the establishment of optimal breeding programmes requires the appropriate use of genetic variation among and within breeds and the judicious use of hormone products, which is an appropriate strategy in developing countries and more developed market economies (Santos et al., 2015). This review is concerned with the use of hormone products in rams to improve reproductive efficiency, which has implications for the productive economics of flocks (Hashem and González-Bulnes, 2021). Scientific articles from the last 50 years, evaluating the use of gonadotropin-releasing hormone (GnRH) in rams, were considered (Arroyo et al., 2016; Cardona-Tobar et al., 2020). This approach differs from the use of "green" techniques and methods that have been promoted to date, such as the use of the male effect and nutritional management in flocks.

## Spermatogenesis in the ram

**Figure 1:**
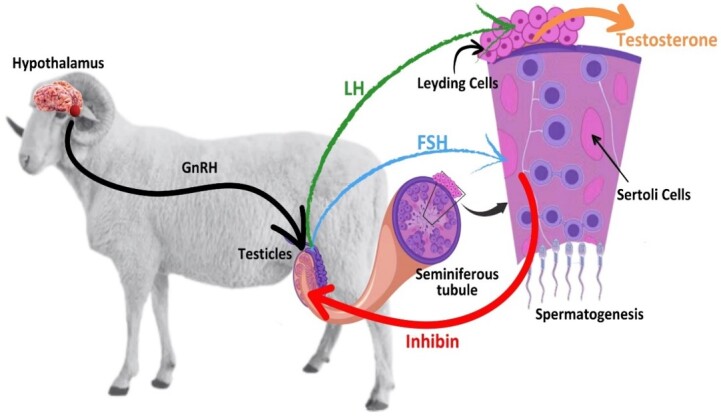
Hormonal regulation of spermatogenesis in rams.

As a preamble to this review, a general description of spermatogenesis in rams is given, followed by a focus on the hormones that have been used in recent years to increase ram reproductive efficiency. Spermatogenesis, the process that transforms germ cells into spermatozoa, is governed by mitotic and meiotic processes, going from cells with 54 chromosomes to 27 (Moraes et al., 1980; Cardoso and Queiroz, 1988). These mechanisms take place in the seminiferous tubules, which protect and support the germ cells and in which the spermatozoa are formed in their entirety, in a process that takes approximately 48 days in rams as five cycles of approximately 10 days each are required (Courot and Ortavant, 1981). The process is divided into three phases, which are regulated by the pituitary hormone system (Figure1). The hypothalamus secretes pulsatile GnRH, which stimulates the anterior pituitary to produce and release gonadotropins: luteinising hormone (LH) and follicle-stimulating hormone (FSH), the main players in the mechanisms of gametogenesis (Cardoso and Queiroz, 1988). As hormonal mechanisms, they are controlled by various factors such as diet, light-dark hours, age, heat stress and thermoregulatory processes (Cardona-Tobar et al., 2020). When released, LH binds to Leydig cell receptors and stimulates the synthesis and secretion of testosterone (T) in the inter-testicular space, binding Sertoli cells (SC) at the nuclear level. As FSH is also released, it will bind to SC membrane receptors and work in conjunction with T to induce Sertoli cells to produce androgen-binding protein (ABP), seminiferous fluid, androgen-binding proteins and hormones. When FSH binds to Sertoli cells, it also stimulates the production of inhibin and activin, exerting negative feedback with the pituitary axis and controlling the secretion of FSH and LH. The FSH is required to initiate spermatogenesis at puberty, but once initiated, it is required to initiate spermatogenesis only when inhibited by external factors (Wang et al., 2022; Ramaswamy and Weinbauer, 2014). At puberty, which occurs in sheep occurs at around 4 months of age, spermatogenesis begins because at this stage, the Leydig cells have already differentiated into adult cells and contain receptors for LH and synthesise and release T, initiating spermatogenesis.

The signalling cascade and the mechanisms that carry out spermatogenesis are affected by a number of factors that severely compromise the ram's reproductive performance. These factors include heat stress, which causes fatigue, and dehydration, which adversely affects ram development and production by activating thermoregulatory mechanisms such as thermal shock, with negative impacts on food consumption and energy requirement by compromising nutrition (Macías-Cruz et al., 2018b, c). Similarly, cell division-induced oxidative stress, which consumes mitochondrial oxygen and makes the ram's reproductive system vulnerable, is also produced by thermal, nutritional and seasonal stress, among others (Asadi et al., 2017). Spermatogenesis begins at puberty, and its onset varies among in months among breeds, with early age being the most favourable for sexual performance. However, although the number of sperm decreases over the years, sperm cells do not disappear completely; notwithstanding, the length of the seminiferous tubules decreases with age, affecting sperm production. In parallel to the above, seasonality also plays a role since when the light-dark hours are affected, the neuroendocrine that causes seasonal anoestrus is affected in most rams (Stewart et al., 2021). In the following section, we look at the hormonal strategies that can be integrated to reduce negative effects on spermatogenesis and improve semen quality in rams.

## Sexual behaviour of rams

Through vast amounts of information presented by different studies involving the analysis of male reproductive hormones, it has been confirmed that the sexual activity of rams is under the control of androgenic hormones that act at the level of the hypothalamus (preoptic area) (see complete reviews of Orihuela-Trujillo, 2012). One of the main hormones released by rams is testosterone, which influences sexual and metabolic activity and varies throughout the year; it is present at low concentrations outside the reproductive season, while testosterone levels increase above the threshold required for the ram to show sufficient sexual behaviour to detect a female in oestrus (Bahadi et al., 2023) and induce her to cover and become pregnant within the same season. Ungerfeld (2021a) systematically characterises sexual behaviour and describes the main scenarios that can influence sexual development from lambing to adulthood. The success of any sheep breeding programme depends on three important reproductive aspects of the ram: libido (interest in mounting a female), mating ability (ability to cover ewes in heat) and semen quality (fertility) (Price, 1987). These three key points are required to ensure that each of the rams involved in a mating programme will have interest in covering an oestrus ewe and will present good semen quality. Similarly, rams with poor sexual libido which show poor competition for an oestrous female will produce fewer offspring in future generations. In this case, the component known as sexual behaviour in rams has been the subject of much research under different scenarios to determine whether it contributes significantly to the effectiveness of the male effect (Perkins and Fitzgerald, 1994). The same authors compared high and low sexual behaviour rams when exposed to Targhee and Ramboulliet wool ewes; they found that a high percentage of ewes ovulated when exposed to high sexual behaviour rams (95%) compared to ewes exposed to low sexual behaviour rams (78%).

**Figure 2:**
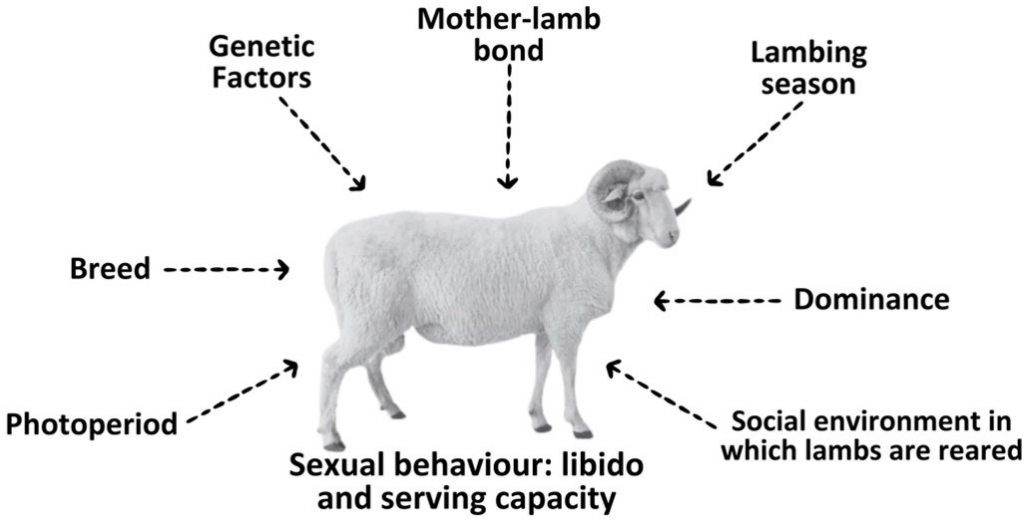
Factors influencing sexual behaviour in rams (as mentioned in the reviews by Orihuela-Trujillo, 2012; Ungerfeld, 2021a, b).

The aim of this review is not to describe all of the factors involved in or influencing sexual behaviour and the male effect, as comprehensive reviews have been previously undertaken over the years (see full reviews by Pretorius, 1972; Tilbrook and Cameron, 1990; Rosa and Bryant, 2002; Ungerfeld et al., 2004; Perkins and Roselli, 2007; Orihuela-Trujillo, 2012; Roselli, 2020; Ungerfeld, 2021a, b). However, it is necessary to mention that consistent studies have been carried out over the years investigating factors that influence sexual behaviour (see Figure 2), where the different factors that can influence both wool and hair rams have been shown (Bahadi et al., 2023). It is important to bear in mind that there are differences which, in one way or another, affect the mating of a ram to an oestrus ewe to a greater or lesser extent, as well as the achievement of optimal gestation of two offspring. Based on these factors, studies have been performed to evaluate different hormones used to improve the sexual behaviour of rams in the different scenarios highlighted in Figure 2.

## Reproductive seasonality of rams

**Figure 3:**
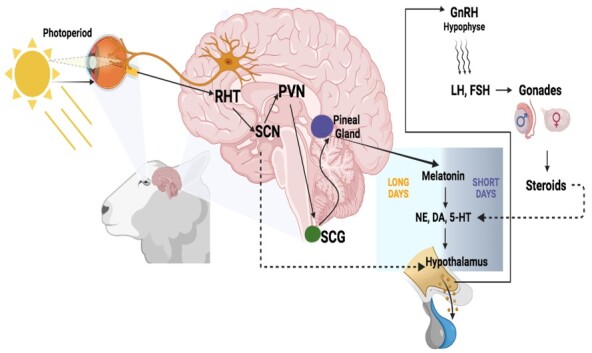
RHT: Retinohypothalamic tract, SCN: Suprachiasmatic nucleus, PVN: paraventricular nucleus, SCG: superior cervical ganglion, PG: Pineal Gland, NA: Noradrenaline, DA: Dopamine, SHT: Serotonin, GnRH: Gonadotropin-releasing Hormone, LH: Luteinising hormone, FSH: Follicle-stimulating hormone.

Among these factors, the one that most influences sexual behaviour is related to seasonality, as most breeds are seasonal and show a higher libido when natural light decreases (short days). This reproductive symphony is controlled by the photoperiod (see Figure 3). In the cited literature, it is confirmed that rams are less affected than ewes, but maintain their fertility (Santos et al., 2015; Zaher et al., 2020; Hedia et al., 2020; Kleeman et al., 2021), especially in hair rams (Sánchez-Dávila et al., 2020; de Sá Geraldo et al., 2024). However, different hormonal strategies have been evaluated to improve the sexual activity of rams in and out of the breeding season, which are described in the following sections.

On the other hand, with regard to the physiological differences that may exist between hair and wool rams from a reproductive point of view, it should be mentioned that the reproductive seasonality is longer and more pronounced in wool rams, and they show a longer seasonality due to their greater body weight, with each breed responding to the photoperiod depending on the latitude at which they are commercially exploited (Ungerfeld, 2012). In this case, woolly males show seasonal variations in semen quality and sexual activity, the duration of which is less pronounced compared to ewes (Mamontova et al., 2021; Aibazov et al., 2022). Consequently, woolly sheep breeds are characterised by different photoreactivity, where the effect of seasonality and circadian variations on sexual behaviour and semen quality in rams has been studied from tropical latitudes to both poles of the earth (Godfrey et al., 1998; Mamontova et al., 2021). On the other hand, with regard to hair rams, recent studies have been carried out on the influence of seasonality on their reproductive physiology, where it was found that they can show variations, such as in semen quality, but not reach zero values, for example in Pelibuey and BlackBelly rams (Aguirre et al, 2007; Cárdenas-Gallegos et al., 2012), Saint Croix rams (Sánchez-Davila et al., 2020) and Santa Inés rams (de Sá Geraldo et al., 2024); all studies concluded that the seasonal dimension was not sufficient to prevent rams from being able to cover and gestate ewes at different times of the year. Similarly, in terms of sexual behaviour, large differences can be found between wool and hair breeds, where wool rams can reach no or low sexual activity (Avdi et al., 2004; Mamontova et al., 2021) compared to hair rams, which are mainly used at latitudes below 22° and show significant variations in sexual behaviour when there are high ambient temperatures and relative humidity (Aké-Villanueva et al., 2019; Sánchez-Davila et al., 2020; de Sá Geraldo et al., 2024). For example, the use of hair rams to stimulate Suffolk wool ewes during the anoestrus period achieved up to 91.66% of ewes being covered and lambed when mounted by Saint Croix rams (Clemente et al., 2012). Therefore, the use of hormonal strategies is currently focused on eliminating the effects of seasonality, mainly in wool rams; in contrast, the aim in hair rams is to minimise the effect of environmental factors in order to increase the reproductive efficiency of males in any mating season.

## Administration of hormonal products to stimulate sexual activity and spermatogenesis in rams

At present, the use of hormonal strategies to improve semen quality and sexual behaviour in rams is mainly based on six of the most important hormones that have been studied to improve the reproductive performance of rams and have a positive impact on flock productivity. The scientific literature was searched for the most representative publications for each of the hormones described in this review. This is the first review that collectively describes the hormones that have influenced the scientific development of ram reproduction over 50 years or more. There is currently a great deal of research, including reviews, on each of these hormones, but they have not previously been described together. In addition to the above productive advantages, hair rams do not exhibit the pronounced seasonal reproduction seen in wool breeds (Macías-Cruz et al., 2009). Although semen quality and reproductive activity are reduced, the ability to mate and produce semen is maintained throughout the year (Stewart et al., 2021). Currently, various hormonal alternatives are being sought to increase sexual activity and semen quality in rams as this is a novel research field and has not been fully explored. Over the past decade, hormones such as cloprostenol (Daham, 2012) and T propionate (Calderón-Leyva et al., 2019), prostaglandin analogues and equine chorionic gonadotropin (eCG), as well as kisspeptin (Daniel et al., 2015), melatonin (Pool et al., 2020b) and oxytocin (El-Shalofy and Hedia, 2021b), have been investigated in rams.

### Kisspeptin (Kp)

Successful reproduction control, at least in ruminants and especially in sheep and rams, requires the integration of external and internal factors so that the pulsatile secretion of GnRH by the hypothalamus can take place; the most important factors are the feedback of sexual steroids, feeding and body condition, breeding season, pheromone production, stress and age of the animals (Scott et al., 2019; Harman and Serpek, 2022). Kisspeptin, also known as metastin, inhibits the metastasis of cancer cells (Daniel et al., 2015). In 1971, it was discovered that the hypothalamic-pituitary-gonadal axis is responsible for regulating reproduction in mammals, but the positive and negative feedback mechanisms that regulate the pulsatile secretion of GnRH were not elucidated until 2003 with the discovery of the set of neurons called kisspeptins, one of the most important discoveries in relation to mammalian reproduction (Asadi et al., 2017; Abou-Khalil and Mahmoud, 2020; Meccariello et al., 2020). Kisspeptin and its receptors also play an important role in the regulation of the reproductive axis; kisspeptin neurons are produced in the preoptic area (POA) and arcuate nucleus (ARC) neurons and act directly on GnRH neurons, stimulating gonadotrope cells to release LH and FSH and acting directly on the male gonads of mammals (Meccariello et al., 2020). Kisspeptin is an important neuropeptide involved in the onset of puberty, the regulation of fertility, the secretion of gonadotropins and the proper functioning of the gonads (Beltramo and Decourt, 2018; El-Sherry et al., 2020). In addition, it is a regulator of the male reproductive system with a wide range of functions; improves semen quality, including sperm viability and concentration, and increases testicular T levels (Abou-Khalil and Mahmoud, 2020; Beltramo and Decourt, 2018). Kisspeptin has been detected in the epididymal epithelium, and its receptor is expressed in the acrosomal region of both spermatids and mature sperm (Abou-Khalil and Mahmoud, 2020). This renders the sperm capable of fertilisation during transfer through the epididymis and into the female reproductive tract (Meccariello et al., 2020). Kisspeptin has also been found in Leydig and Sertoli cells (Beltramo and Decourt, 2018). Spermatids and mature spermatozoa possess a receptor for kisspeptin and are exposed to kisspeptin-synthesising cells from the seminiferous tubules to the female reproductive tract. Thus, kisspeptin is part of the microenvironment of spermatozoa and mediates their maturation (Abou Khalil and Mahmoud, 2020).

**Figure 4:**
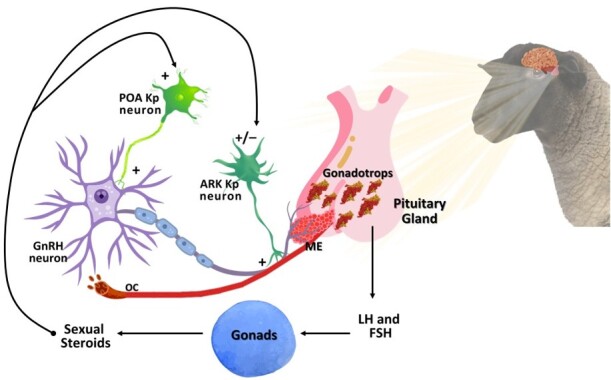
Schematic representation of the mechanisms controlling reproduction in rams.

However, especially in zootechnically important animals, endogenous Kp has limitations in terms of its short half-life and poor pharmacodynamics, limiting its use in large-scale experiments (Seminara, 2006). Recently, synthetic KISS1R agonists based on the kisspeptin scaffold have been developed, which enabled studies on their use. For example, EL-Sherry et al (2020) which showed that the intramuscular application of 5 µg/kg live weight of Kp in Ossimir rams increased the ejaculate volume, sperm concentration and percentage of live sperm. Similarly, the trajectory and rheotaxis of spermatozoa were improved by the use of Kp, which appears to influence the neuroendocrine control of reproduction, at least in ruminants, culminating in the secretion and metabolism of LH (Daniel et al., 2015). Kisspeptin is encoded by the KISS1 gene and acts through GPR54 (Kiss1r) to induce GnRH secretion (Seminara, 2006). Because of its importance in the brain, Kiss1r is expressed by GnRH neurons. Administration of Kp to ruminants stimulates the release of LH and FSH (see Figure4) (Harman and Serpek, 2022; Abou-Khalil and Mahmoud, 2020; Abdel-Ghani and Mahmoud, 2019). The intra-testicular effect of Kp on T production and spermatogenesis in ruminants is the subject of ongoing research.

Abou Khalil and Mahmoud, 2020 mention that treating rams aged 1.5–2 years with a dose of 5 mg/kg body weight of kisspeptin-10 (KP-10)/week for 1 month improves testicular weight, which depends on sperm development and may be highly related to T concentration. This leads to an improvement in the histological characteristics of the testes caused by the proliferation and differentiation of KP-10. In the same experiment, treatment with KP-10 had a direct effect on the significant improvement in semen quality, including scrotal circumference. In studies in prepubertal cattle, Kp stimulated the secretion of LH and FSH (Ezzat et al., 2010), and in monkeys, it stimulated the secretion of LH, FSH and T (Ramaswamy and Weinbauer, 2014). In a more recent study carried out in bucks, where kisspeptin-10 was evaluated at doses of 4 and 8 µg/kg/animal outside the reproductive season, the T concentration increased 50 minutes after the application of both doses (Al-Ameri et al., 2019). In a study by Rietema et al. (2019), more protein cells were found in the mid-dorsomedial hypothalamus and mid-arch when the rams were sacrificed, suggesting that they mediate the increase in GnRH and LH production due to acute nutritional supplementation, which may be more pronounced when rams are given acute protein supplementation (30 g kg-1 bw per day) as it activates Kp neurons in the arcuate nucleus of the brain.

### Oxytocin

Oxytocin (Ox) was first described as a uterotonic agent in 1906 and first synthesised in 1953 (Stadler et al., 2020). It is a hormone that is routinely used on dairy farms (Rietema et al., 2019). Oxytocin is stored and released by the posterior pituitary of the hypothalamus and is a peptide neurohormone; its receptors are found in the brain, pituitary gland, heart and gonads (Rietema et al., 2019; Stadler et al., 2020; Knickerbocker et al., 1988). This hormone contracts the smooth muscle of the uterus and mammary gland, which is important for sperm transport after copulation as well as facilitating labour and udder contractions (Stadler et al., 2020). Similarly, Ox is produced by Leydig cells, the epididymis and the prostate, where its receptors are found (Knickerbocker et al., 1988), and increases the contractions of the seminiferous tubules and the walls of the epididymal ducts, thus facilitating the transfer of spermatozoa (Stadler et al., 2020; Assinder et al., 2000; Dalmazzo et al., 2019; Ruan et al., 2020). It is frequently used today, especially in male reproduction in small ruminants, in different aspects, such as for semen collection with the electro-ejaculator, where, when used together with the prostaglandin (PG) before semen collection, it reduces the time and number of electrical stimuli required to obtain a semen sample without affecting its quality, both in goat bucks (Ruan et al., 2020) and rams (Abril-Sánchez et al., 2019; Luna-Palomera et al., 2021).

Considering that Ox increases sperm transport by increasing epididymal contractility and that is an effective and economical adjunct to improve at least one important characteristic of semen quality, (Stadler et al., 2020; Luna-Palomera et al., 2021), a dose of 10 IU of this hormone can increase the ejaculate volume in semen collection in rams using an artificial vagina. However, it is important to note that equally important characteristics such as sperm concentration, motility and serum T concentration were not affected, which relegates this treatment to an option to increase semen volume only.

Oxytocin is produced locally in Leydig cells and is under the control of LH and lipoproteins but not T (Stadler et al., 2020). It regulates basal epididymal contractility and stimulates sperm release from the storage site in the epididymis at the time of ejaculation (Knickerbocker et al., 1988; Dalmazzo et al., 2019; Stadler et al., 2020). Application of Ox prior to semen collection can increase sperm concentration in several mammalian species including rams, rabbits, cattle and buffalo (Luna-Palomera et al., 2021). Therefore, it seems reasonable that Ox is involved in the transition of sperm through the male ducts and may accelerate ejaculation (Knickerbocker et al., 1988; Abril-Sánchez et al., 2019). In this sense, Ox can help improve semen quality. In rats and sheep, exogenous Ox administration affects the contraction of the seminiferous tubules to promote the exit of sperm towards the rete testis (Da et al., 2013; Stadler et al., 2020). Another study showed that Ox can regulate basal epididymal contractility while stimulating sperm release during ejaculation (Luna-Palomera et al., 2021).

In rams, exogenous Ox application can increase the concentration of spermatozoa (Assinder et al., 2000). This has been verified by immunocytochemical studies: when a dose of 20 µg of Ox was applied to adult rams, Ox receptors could be identified in Leydig and Sertoli cells as well as in the epithelial cells of the epididymis, thus confirming that Ox can modulate sperm transport, which is supported by the location of Ox receptors in the tail of the epididymis and the *vas deferens*, thus regulating the steroidogenesis of the ram (Jung and Yoon, 2021). However, at least in male goats, administration of 0.7 IU/kg Ox increased testicular blood flow (vasodilator effect) and decreased plasma T concentrations (control of stereodigenesis) 60 minutes after application, which can be explained by the inhibition of the formation of the C21 and C19 metabolites of pregnenolone and, therefore, T concentrations (Hedia and El-Shalofy, 2022). These differences may be partly due to the different species and doses studied. Regarding seminal quality in rams, the administration of low doses of Ox (5 IU) increased the volume, motility and sperm concentration at 10 minutes after application (Narenji Sani et al., 2014). In males of other domestic species, such as rabbits (Fjellstrom et al., 1968), cattle [(Berndtson and Igboeli, 1988) and buffaloes (Ibrahim, 1988), there is also evidence of a positive effect of Ox on semen quality (Tiptanavattana et al., 2022). In rams, however, it must be considered that the contractile effect of Ox would lead to the emptying of the spermatozoa stored in the epididymis, resulting in an increase in the number of abnormal spermatozoa after the 6th week, which is the period required for spermatogenesis (Stadler et al., 2020). A study in Mexico evaluated the effect of the administration of exogenous Ox prior to semen collection with an artificial vagina in Pelibuey rams (Luna-Palomera et al., 2021); the application of 10 IU intramuscularly significantly increased the semen volume by 30% compared to that of rams not treated with Ox, but without a significant effect on semen quality variables such as total sperm concentration and sperm per mL. Another aspect evaluated in this work was the effect of 10 or 20 IU of Ox 20 minutes after administration on T levels, with no significant differences found. The results described above contrast with those of previous studies (Nozdrachev et al., 1994; Inaba et al., 1999) that investigated the relationship between exogenous Ox and other hormones. However, the effects of Ox on T are still subject of debate as some authors reported that Ox treatment reduces the T concentration (Berndtson and Igboeli, 1988) or has no impact on it (Ibrahim, 1988), whereas others (Nozdrachev et al., 1994) report that it increases T release. In terms of sexual behaviour, the application of 10–20 IU of Ox in goats has a positive effect on libido and reduces the time needed to collect semen samples (Çiğdem et al., 2019). This is due to the interaction of components involved in male sexual behaviour, such as dopamine, serotonin, amino acids and Ox, which act directly on the paraventricular nucleus of the hypothalamus (Harris and Nicholson, 1998; Argiolas and Melis, 2004; Bozkurt et al., 2007). In a descriptive way, Table 1 presents the most recent summarised studies on the use of Ox in rams.

**Table 1:** Summary of the studies on oxytocin, including breeds, doses, method of administration, time of the study and the main effects.

**Reference**	**Breeding season**	**Breed**	**Application method**	**Collection method**	**Collection time after application**	**Dose**	**Main effects**
El-Shalofy and Hedia (2021a)	Within	12 Baladi goat bucks	IV	Does not apply	Does not apply	0.7 IU/kg at 0, 5, 30, 60 and 120 minutes	Testosterone decreased 60 minutes post-treatment. Oestradiol 17-β not affected. Increased testicular blood flow
El-Shalofy and Hedia (2021b)	Within	16 Ossimi rams	IV	Does not apply	Does not apply	Single dose of 20 IU	Testosterone and oestradiol-17β increased from 5 to 30 minutes after injection and decreased until 120 minuates. Increased testicular vascular tone
Nicholson et al. (1999)	Not reported	24 Oxford Down rams	IV	Cannulation of the epididymidis	Each 10 min	10 and 100 μg	Increase in the number of spermatozoa and seminal fluid. Promoted an increase in the transport of spermatozoa in the *vas deferens* and the ejaculate
Whittington et al. (2001)	Within	15 Oxford Down rams	IM	Castration of the rams after application	After 24 hours, semen samples were extracted from the testicles	Single dose of 20 μg	Increased the concentration of spermatozoa in the rete testis
Çiğdem et al. (2019)	Within	20 Norduz bucks		Artificial vagina		10 and 20 IU	Collecting the semen sample 20 minutes after the administration of 20 IU oxytocin did not improve overall semen quality. However, libido, semen volume and sperm concentrations were increased

It shows the main values obtained from each of the studies cited; 62.5% of these studies were carried out during the reproductive period, mostly in hair breeds. Regardless of the application route and the doses used prior to semen collection, it is standardised that Ox has a main effect on ejaculate volume and increases the T concentrations (Anjum et al., 2018). This is especially true in the first 10 min after application, regardless of the route of administration (IM or IV). Another interesting point is that there are positive effects on ejaculate volume and T levels, regardless of the method of semen collection (Stadler et al., 2020).

In recent years, Ox has been used in combination with PGF2α to obtain semen samples by the electroejaculation technique and to reduce the stress linked to this technique (Palmer et al., 2004; de Moura Fernandes et al., 2021). In this sense, Ox modulates the contractility of the ram's reproductive tract and affects sperm transport and maturation as well as spermiation (Assad et al., 2016). For example, in a study conducted in male goats, the combined use of a PGF2α analogue (cloprostenol, 250 μg IM) and Ox (10 IU IM) 5 minutes and 30 seconds before electrical stimulation respectively, decreased the number of pulses and tended to decrease the number of vocalisations presented by the goat bucks receiving this treatment; seminal quality was not affected (Ungerfeld et al., 2018). The same effect was reported in rams treated with the same doses of PGF2α and Ox analogue, where the administration of GnRH (4.2 μg of buserelin acetate) increased the T levels (de Moura Fernandes et al., 2021). Similarly, another recent study using an Ox analogue (carbetocin, 0.1 mg) via IV found limited effects on the time required for semen sample collection in bucks and only an effect on respiratory frequency in Iberian ibex (Ungerfeld et al., 2023). These new studies on the use of Ox alone or in combination with PGF2α require further studies on the frequency and dose, also outside the reproductive season, which means that the males might show high semen quality but without sexual behaviour.

### Gonadotropin-releasing hormone (GnRH) agonists

The GnRH is a hormone produced by neurons in the arcuate nucleus of the hypothalamus. It has an endocrine function and is transported via the portal system to the pituitary gland, activates its own receptors and stimulates the release of FSH and LH via the pituitary gland so they can perform their functions in the ovaries and testes (see Figure 3) (Adams, 2005; Zhao et al., 2023). In general terms, GnRH is a decapeptide comprising 10 amino acids arranged sequentially. It is a neurohormone as it is produced by neurons. The gene encoding the GnRH precursor is situated on Chromosome 8 in mammals (Foster et al., 2006; Zhao et al., 2023), and GnRH is secreted in pulsatile form and released into the portal system in pulses of different amplitudes and frequencies. Its half-life is short (2–4 minutes) (Zhao et al., 2023). In both goats and sheep, high GnRH activity levels commence during puberty and continue throughout the reproductive lifespan (Foster et al., 2006).

As a result of identifying the activity of GnRH, comparable molecules, known as analogues, have been developed synthetically. To imitate the effect of GnRH and to bond tightly with its receptors in the pituitary gland, these molecules replace some amino acids within the initial GnRH molecule (Moenter and Evans, 2022). The GnRH analogues are capable of obstructing the natural GnRH activity and hindering the release of FSH and LH from the pituitary gland to the gonads (Adams, 2005). This function has been leveraged to create medications for use in assisted reproduction therapies, artificial insemination and *in vitro* fertilisation (Hawken and Martin, 2012).

Two types of GnRH analogues have been developed, with different mechanisms of action. The first type are GnRH agonists, which have a longer half-life and a high affinity for the GnRH receptor. Binding of the agonist to the receptor results in an abrupt release of gonadotropins, known as the "flare-up" effect, which is a sudden increase in LH release and used in reproductive programmes to synchronise ovulation (Moenter and Evans, 2022). However, a drawback of these GnRH agonists is that with continuous administration, the receptors become obstructed and unresponsive, resulting in the suspension of FSH and LH secretion (Adams, 2005). However, GnRH antagonists are more directly inhibitory in their effect as they block the receptor immediately upon binding (Amodei et al., 2022; Hitesh and Kansal, 2022; Hashem et al., 2023). Moreover, in goat and sheep production systems, farm productivity must be based on the continuity of kidding and lambing throughout the different seasons. Therefore, since the 1980s, the priority for both species has been to increase the number of continuous births and to improve lamb and kid yield as well as milk production. To this end, natural strategies such as light programmes for males and the use of melatonin implants have been developed, based on the knowledge of the male effect in both species, to significantly improve economic indices outside the non-breeding season. Although these techniques are effective, the males take time to respond. In this case, as with the previous hormones, GnRH agonists have been used in zootechnically important males to release T (Do Espìrito Santo et al., 2022, Yousef et al., 2022). In females, their use has been extended to oestrus and ovulation synchronisation programmes as well as multiple ovulation and embryo transfer (MOET) programmes. However, they are now starting to be used in small ruminant males as there is not sufficient information on females (Prestel et al., 2022).

In the late 1960s and 1970s, the application of GnRH to enhance semen quality and stimulate T release in rams was first explored. Schanbacher and Lunstra (1977) evaluated the use of 50 µg twice daily for 7 weeks and provided evidence of an advantageous effect on sexual behaviour, as measured by a mating index score, with 60% of the rams treated with GnRH successfully completing the 20-minute behavioural test. In a recent study conducted in bucks, the intramuscular administration of a GnRH analogue (buserelin acetate = 4.2 µg/animal/10 days) resulted in a significant increase in T concentrations, particularly in the initial hours after administration. Improvements were also observed in certain semen quality variables such as mass motility as well as the percentages of motile and normal sperm. It was concluded that, outside of the reproductive season, the short-term administration of buserelin increases T levels and enhances sperm quality without impacting the secretion of gonadotropins from the pituitary-gonadal axis and, consequently, the T levels (Giriboni et al., 2019). Another study indicated that, at least in dogs, using GnRH could rectify sperm dysfunction through subcutaneous administration of three injections. These should be administered once per week at a dose of 1 µg/kg of buserelin acetate. However, it should be noted that the extended usage of GnRH inhibits LH and T secretion by the anterior pituitary and testes (Kawakami et al., 2005). A strong effect of GnRH occurs shortly, after the first hours of application.

In a study conducted on Ossimi rams, applying a single dose of GnRH (buserelin acetate: 0.008 mg/ram) intravenously resulted in an increase in T levels during the first 3 hours and a progressive increase in oestradiol-17β levels from the 3rd to the 120th hour of application (El-Shalofy and Hedia, 2021a). However, it should be noted that the excessive use of the agonist buserelin acetate may result in negative feedback effects. In Santa Inés rams, the T levels peaked at week after administering 2.5 µg of buserelin acetate (Do Espírito Santo et al., 2022). Additionally, in bulls, applying 100 µg/48 h can preserve the scrotal thermoregulatory system's function without impacting semen quality (Romanello et al., 2018). These studies have demonstrated that although the LH secretory responses decreased after single or repeated injections of GnRH, maturation of the testes' steroidogenic capacity occurred. The increased steroidogenic activity may have been a direct result of the increased number of Leydig cell receptors (Wilson and Lapwood, 1979).

To summarise the action of GnRH in the regulation of reproduction, the animal's own intrinsic and environmental factors influence the hypothalamic-pituitary-gonadal axis, resulting in the pulsatile secretion of GnRH from the hypothalamus. Therefore, in view of the global legislative requirements to provide better animal welfare, it should be taken into account that the increasing use of more natural diets and such that affect the nutritional status of animals and limiting the use of steroid hormones in reproductive processes and the secretion of FSH and LH to regulate endocrine function and increase ram reproductive efficiency are important measures. It is therefore certain that the prolonged use of GnRH agonists will significantly reduce serum T concentrations, testicular volume and spermatogenesis Adams (2005), which confirms the current importance of research aimed at determining the optimal dose of GnRH agonists in rams under different environmental scenarios, with emphasis on nutrition, age and breed, among other factors.

### Equine chorionic gonadotropin (eCG)

eCG is a high molecular weight glycoprotein derived from the endometrial cups of the mare's uterus (produced from 35-100 days of gestation); it is extracted from the blood of pregnant mares. In these females, eCG has LH activity (Luna-Palomera et al., 2019), but in the cow it has activity as FSH or LH, depending on the receptor populations in the ovarian follicles at the time (De Rensis and López-Gatius, 2014; Murphy, 2018; Bo and Menchaca, 2023). Regarding eCG in rams, recent studies conducted by a group of researchers from South America (Uruguay) evaluated the application and evaluation of eCG in small ruminants (bucks and rams) under different scenarios. Their research was published in high-impact journals, giving validity to the results obtained. Table 2 provides a summary of the work carried out mainly by this group. According to the results, the use of eCG within and outside the breeding season affects T concentrations and sexual behaviour but not semen quality and scrotal circumference. In rams and bucks, it has been used since 2008, when doses of 100 to 400 IU of eCG were evaluated in prepubertal lambs (Ungerfeld and Bielli, 2008), with no effect on semen quality and sexual behaviour; However, at does higher than 400 IU, up to 1000 IU, or when two doses of 1000 IU are given to rams 3 days before mating with ewes, it can induce an increase in T secretion in rams during the period of sexual rest (Ungerfeld et al., 2018; Ungerfeld et al., 2019), but without altering semen quality (Beracochea et al., 2020). In this sense, eCG has been used to increase libido and make the use of the male effect more efficient in rams (Ungerfeld et al., 2014, 2019). Similarly, its use has been extended to bucks, as in rams, but with an initial dose of 800 IU of eCG and four doses of 500 IU applied every 5 days during the period of sexual rest, resulting in an increase in T secretion and an improvement in the quality of fresh semen, with an increase in the level of eCG antibodies (Beracochea et al., 2018).

**Table 2:** Summary of the studies on eCG, including breeds, doses, method of administration, time of the study and the main effects.

**Reference**	**Breeding season**	**Breed**	**Application method**	**Collection method**	**Collection time after application**	**Dose**	**Main effects**
Narenji Sani et al. (2014)	Outside	18 Zel rams (Iran)	IM	Electroejaculator	10 minutes	Single dose 0, 400 IU, 600 IU	Increased semen volume, mass activity, total number of sperm
Ungerfeld et al. (2018)	Within	20 Saint Croix rams	IM	Does not apply	Does not apply	**First study:** Two doses of 1,000 IU of eCG on days 0 and 4	Testosterone concentration increased in rams given eCG both within and outside the breeding season. Better sexual behaviour within the breeding season in rams given eCG.
	Outside	10 Saint Croix rams	IM	Does not apply	Does not apply	**2nd study:** Two doses of 1,000 IU of eCG on days 0 and 4	
Beracochea et al. (2018)	Outside	19 Gabon bucks	IM	Electroejaculator	-14 to 56 days	Initial dose: 800 IU (day 0), followed by four doses of 500 IU every 5 days	Administration of eCG affected testosterone concentration, antibody titres, the percentage of motile spermatozoa and spermatozoa with progressive motility and the percentage of spermatozoa with a functional membrane throughout the study period
Beracochea et al. (2020)	Outside	19 Gabon bucks	IM	Electroejaculator	91 days	Initial dose: 800 IU (day 0), followed by four doses of 500 IU every 5 days	The anti-eCG titre was higher in treated bucks (181.7 ± 61.3 ng/µL) vs control bucks (31.1 ± 10.7 ng/µL). No significant differences among treatments in testosterone concentration, fresh and thawed semen characteristics, scrotal circumference and sperm cryoresistance
Beracochea et al. (2020)	Outside	27 Merilin rams	IM	Electroejaculator	-4, -1, 3, 6, 9, 12, 15 and 22 days	**First study:** Three doses every 6 days: 0, 400 and 700 IU. Day 0 = day of the first administration.	Testosterone concentrations were statistically higher in rams treated with 700 IU eCG than in rams treated with 0 or 400 IU eCG. Testicular characteristics and semen quality did not differ among treatments
		15 Highlander; 17 Texel	IM	Electroejaculator	-7, -5 and weekly for 3 weeks	**Second study:** two doses of 1,000 IU of eCG: days 0 and 5	The use of eCG had no effect on scrotal circumference and fresh semen characteristics. The ratio of cryopreservation of motile and progressive spermatozoa was higher in rams treated with eCG compared to the control group
Abbas et al. (2021)		9 Beetal bucks	Subcutaneous and IM	Artificial vagina		T1 = melatonin implant of 18 mg and 400 IU of eCG (18 injections every 4 days)	Testosterone concentration increased from week 3 for T1 (4.2 ± 0.2), followed by T2 (1.2 ± 0.1) and T3 (0.28 ± 0.1). Melatonin levels also increased at week 5 in T1 (12.5 ± 0.2), followed by T2 (10.2 ± 0.1) and T3 (3.89 ± 0.2) (p < 0.05). Sperm progressive motility and kinematics, acrosomal and DNA integrity improved from T1 to T2 and T3 (p < 0.05)
						T2 = melatonin implant alone and T3 = control	

Based on the literature, eCG has an effect on the increase in T concentration, and only two articles mention that it has an effect on seminal quality. Specifically, Narenji Sani et al. (2014), using Zel rams and eCG doses of 400 and 600 IU, and Beracochea et al. (2020), reported increased cryopreservation rates (see Table 2). Notably, it is difficult to obtain high-quality semen from rams treated with eCG as it has a half-life in the blood of 63 hours due to its high content of sialic acid, a sugar that is incorporated into eCG proteins during glycosylation in cells and acts as an intercellular signalling agent (Aguirre-Zapata et al., 2023). Recent studies have used the combined application of eCG (400 IU) with other hormones, such as melatonin (18-mg melatonin implant), for 18 weeks in male goats. Based on their results, there was a decrease in non-viable and morphologically abnormal spermatozoa and an increase in T concentrations and semen quality, observed from the 3rd week of the study (see Table 3). (Abbas et al., 2021). These results are interesting because of the combination of the two hormones: on the one hand, eCG, which can directly increase the level of intra-testicular T, directing its action to the differentiated spermatozoa found in the epididymis and in the accessory sex glands, where the eCG receptors are located (Min et al., 2004), causing a change in the composition of the seminal plasma (Matsuoka et al., 2006); on the other hand, melatonin, which has the limitation that it can exert its effect only up to 35 days after its application (Abbas et al., 2021). Therefore, its use may be limited in terms of the frequency of application to have a positive and lasting effect on fertility in rams; however, it may be limited by the production of antibodies, although this does not appear to be the case, at least in male goats (see Table 2) (Beracochea et al., 2018).

**Table 3:** Summary of the studies on PGF2α analogues, including breeds, doses, method of administration, time of the study and the main effects.

**Reference**	**Breeding season**	**Breed**	**Application route**	**Collection method**	**Collection time after application**	**Dose**	**Main effects**
Masoumi et al. (2011)	Not reported	Holstein bulls	IM	Artificial vagina	30 minutes	250 mg cloprostenol	Increased semen volume, spermatozoa concentration and testosterone level in low-libido bulls
Maatoq et al. (2015)	Within	5 buck goats	IM	Artificial vagina	24 hours	7.5 mg cloprostenol 24 hours before semen collection for a period of 5 weeks	Increased individual motility and sperm concentration, decreased abnormal sperm
Olfati et al. (2013)	Outside	20 Crossbred rams with Merino	IM	Artificial vagina	30 minutes before semen collection twice a week/5 week	7.5 mg cloprostenol	Increased semen volume, sperm concentration, total number of spermatozoa per ejaculate
Ungerfeld et al. (2020)	Within	26 Saint Croix rams	IM	Does no apply	5 minutes before sexual tests	10 mg dinoprost tromtehamine	No positive effects on sexual behaviour in rams
Sánchez-Davila et al. (2022)	Within	24 Katahdin rams	IM	Artificial vagina	5 minutes before sexual tests	10 mg dinoprost tromtehamine	Animals in the dinoprost-treated group mated more times than those treated with cloprostenol. Cloprostenol-treated animals had a higher mating efficiency (ejaculations/total matings). Cloprostenol-treated animals produced more semen with better mass motility
					20 minutes before semen collection	0.15 mg cloprostenol dextrogy	

### Prostaglandins

The use of PGs started in the last century (1930), when it was observed that the uterus contracted in response to human seminal plasma (Capitan et al., 1990). This led to a wave of research and extensive literature, with the recent discovery of PGF2α. Today, prostaglandin F2α analogues are hormones used extensively in farm animals to control and synchronise the oestrous cycle in females, particularly in the luteal phase, due to their effects on the ovary, causing luteolysis (Fierro et al., 2013). Prostaglandins are bioactive molecules derived from arachidonic acid. Their biosynthesis is initiated by the action of cyclooxygenase (COX) (Frungieri et al., 2018), which converts arachidonic acid to PGG2, which is further reduced to PGH2 by a peroxidation reaction also catalysed by COX (Echeverrìa, 2006). Two commercial analogues are commonly used experimentally in domestic animals. One is cloprostenol, which was synthesised in 1970 as part of a project to develop PG analogues with potent luteolytic activity (Ramìrez et al., 2018). The other is dinoprost tromethamine, developed in the late 70s of the 20^th^ century. The main characteristic of this substance is that it is similar to that produced naturally in the uterine tract (Moreira and Hammon, 2012; Ramìrez et al., 2018). It is a white salt, crystalline in appearance and hygroscopic in nature, with effects similar to those of PGF2α, in particular luteolytic and vasoconstrictive effects, and is rapidly metabolised by the respiratory system after a few minutes (Sakamoto et al., 1995).

The mechanism of action of prostaglandins is intrinsically linked to specific receptors; these receptors activate a specific G-protein which triggers the AMPc cascade and the corresponding release of calcium via phosphatidylinositol (Echeverrìa, 2006). They are located on the cell membrane and classified according to the type of prostaglandin they bind.

To date, nine receptor types have been identified, namely DP1-2, EP1-4, FP, IP and TP, referring to the receptor that binds the corresponding prostaglandin, with FPs binding to PGF2α (Grösch et al., 2017). Several authors have reported a beneficial effect on ejaculate volume and sperm concentration in dogs (Traas and Root-Kustritz, 2004), bulls (Titiroongruang and Tummaruk, 2011) and bucks (Daham, 2012), which can be explained by the movement of sperm from the epididymis into the *vas deferens* as a result of the aforementioned vasoconstrictive effects.

The mechanisms of action of prostaglandins on male sexual behaviour are not fully understood, but to date, various authors have proposed hypotheses in other species, such as pigs, indicating that PGF2α stimulates some parts of the central nervous system or at least interacts with the hypothalamus, which is one of the regulators of the central nervous system (Estienne, 2014). In cattle, they have been associated with a positive feedback effect on the hypothalamic-pituitary-testicular axis (Sakamoto et al., 1995), and in rodents, they are involved in germ cell development and steroidogenesis (Titiroongruang and Tummaruk, 2011). At the physiological level, in studies on yearling bulls, serum T levels doubled when PGF2α was administered and remained at high levels for a period of 4 hours (Titiroongruang and Tummaruk, 2011). It has been suggested that PGF2α directly stimulates testicular steroidogenesis through the production of cyclic AMP (Estienne, 2014; Grösch et al., 2017). To date, and in the absence of more detailed studies on the effects of PGF2α, it is believed that it directly acts on the contractile tissues from the testicular capsule and epididymis to the *vas deferens*.

The use of PGF2α analogues prior to collection can optimise the number of spermatozoa, as shown in Table 3, where two analogues have been used in recent years, namely cloprostenol dextrogy and dinoprost tromethamine. Regardless of the breed, species and doses used, there is a positive effect on ejaculate volume, mass motility and sperm concentration in goats, bulls and rams. However, due to the consistent lack of studies on rams, few studies have investigated the use of these analogues and their effects on sexual behaviour. For example, regarding the use of PGF2α analogues in hair rams, Ungerfeld et al. (2020) reported that the administration of 10 mg dinoprost tromethamine in adult Saint Croix rams had no positive effects on the main variables of sexual behaviour, which limits their application. In another study (Sánchez-Davila et al., 2022) conducted in Katahdin lambs with no previous sexual experience, the use of PGF2α analogues had a positive effect on sexual behaviour by reducing the time to initiate courtship and increasing the frequency of anogenital sniffs, flehming and lateral approaches (Table 3). Specifically, animals treated with dinoprost had a higher number of mounts, whereas those treated with cloprostenol had a higher mounting efficiency, requiring fewer mounts to ejaculate. The same study reported that the effects of D-cloprostenol on semen quality were highly relevant as the group treated with this drug obtained more volume and greater mass motility per ejaculate than their counterparts treated with dinoprost and the control group; there was also an upward trend in sperm concentration in this group (Table 3) (Sánchez-Davila et al., 2022).

Although the process by which PGF2α acts in the male is not well understood, it has a potential impact on the hypothalamus-pituitary-testicular axis (Borg et al., 1992). Another hypothesis is that in the boar, there is direct or indirect stimulation of some areas of the nervous system, which would cause an increase in sexual appetite (Estienne et al., 2004).

Other recent studies have used synthetic PGF2α analogues in combination with Ox to make semen collection by an electroejaculator more efficient and allow the collection of adequate semen samples. For example, Ungerfeld et al. (2018) evaluated the combined administration of 250 µg of cloprostenol and 10 IU of Ox (5 min and 30 s before the start of semen collection, respectively) in male goats. Based on their results, the application of both hormones prior to semen collection shortened the collection time and reduced the intensity of the electrical stimulation required for semen sample collection, thus improving animal welfare without affecting semen quality.

Notably, given the still limited use and lack of studies on the use of PGF2α in rams, further studies are needed under different scenarios such as breeds, breeding seasons and doses, especially determining whether it has a short half-life and a residual effect after its application for certain periods such as days, weeks or months (personal communication, Ungerfeld et al., 2023).

### Melatonin

Melatonin (5-methoxy-N-acetyltryptamine) is one of the most studied and used hormones in goat reproduction worldwide. It was first described in the bovine pineal gland in 1958 (Wurtman, 1985). In the early 1980s, scientists started to elucidate how melatonin is regulated by the photoperiod and its effect on testicular activity in rams (Almeida and Lincoln, 1984; Yu and Reiter, 1993). During this decade, research focused on the effect of the photoperiod when rams were exposed to artificial light cycles of short and long days, with most studies reporting a circadian mechanism and its correlation with melatonin blood concentrations, photoperiod and reproductive status of rams (Lincoln et al., 1981). In these studies, it was hypothesised that the response of rams to a change in day length depends on ocular photoreception and is dictated by the combined activities of two brain areas, the suprachiasmatic nucleus (SCN) and the pineal gland. At that time, the function of melatonin action in ram reproduction started to be described (Lincoln et al., 1981; Ebling et al., 1988; Hanif and Williams, 1991; Fitzgerald and Stellflug, 1991) (see Figure 3).

Subsequently, in the 1990s, a line of research began to investigate the use of melatonin implants in both sheep and rams due to the discovery of the action of melatonin through the photoperiod (Fitzgerald and Stellflug, 1991; Webster et al., 1991; Lincoln and Maeda, 1992), where rams treated with melatonin implants during a long photoperiod showed stimulated testicular development through a pathway similar to that exerted by endogenous melatonin through the hypothalamus. From this point on, a cascade of investigations was developed regarding the main factors that could contribute to an interaction with melatonin implants, such as breed, diet, dose and implantation period, including the evaluation of semen quality and sexual behaviour in rams (Hanif and Williams, 1991; Rosa et al., 2000).

In the following decade (2000–2010), the presence of melatonin in seminal plasma and its antioxidant effects were found to be highly correlated with the activity of the three antioxidant enzymes superoxide dismutase, glutathione reductase and catalase (Casao et al., 2010a). More recent studies (from 2011 to date) have demonstrated direct effects of melatonin on ram spermatozoa, reducing apoptotic effects and modulating sperm capacitation to improve fertilisation rates (Shayestehyekta et al., 2022; Kumar et al., 2022). This was the guideline for the development of studies on the addition of different concentrations of melatonin to diluted semen to improve its quality in the freeze-thaw process since free radicals (ROS) are formed at this stage (Rizkallah et al., 2022), which have an oxidative effect when they accumulate excessively, altering the sperm cell membrane, resulting in a reduction in sperm motility and viability. This can be explained by the fact that spermatozoa are rich in unsaturated fatty acids and lose most of their cytoplasm during maturation, exposing them to the effects of ROS (Moradi et al., 2020). In addition, more targeted studies have examined the effect of melatonin on testicular haemodynamics (blood flow), which is important for providing oxygen and nutrients to the testes and for eliminating metabolic waste (Shahat et al., 2022a; El-Shalofy et al., 2022).

Originally thought to be secreted only in the pineal gland, we now know that melatonin is produced in other tissues throughout the body, particularly in the male reproductive tract (Webster et al., 1991). As a multifunctional hormone, it is involved in various physiological processes in mammals, such as cardiovascular regulation, immunomodulation, circadian rhythms and, in some species, the regulation of the reproductive season (Gonzalez-Arto et al., 2017; Abecia et al., 2020). It is one of the key hormones in sheep reproduction (Gonzalez-Arto et al., 2017) and secreted in greater amounts by the pineal gland during long nights (Figure3) (Abecia et al., 2020; Jiang et al., 2022). This hormone sends signals that regulate the seasonality of reproduction; in male sheep, it exerts specific effects on the hypothalamus, triggering, by positive feedback, the pulsatile release of gonadotropins, thus establishing control over sexual activity (Tsantarliotou et al., 2008; Harman and Serpek, 2022).

Melatonin has two mechanisms of action: 1) receptor-mediated action, namely the control of seasonal reproduction and modulation of the sleep cycle, involving cAMP and/or phospholipase C as cellular second messengers (Guerrero et al., 2007; Reiter et al., 2009). Therefore, melatonin acts on the control of GnRH secretion and, consequently, on LH and FSH release, affecting testicular activity and T release (Figure 3) (Reiter et al., 2007; Bronson, 2009; Yu et al., 2018), where T secretion is mainly dependent on cAMP signalling, which is stimulated by LH (Gonzalez-Arto et al., 2016). When Leydig cells are exposed to melatonin, T release and cAMP production are increased depending on the dose of melatonin used (Li and Zhou, 2015); 2) receptor independence, which is a characteristic of melatonin due to its liposoluble and water-soluble molecular nature, allowing it to reach all subcellular compartments at physiological concentrations. Based on the above, when exogenous melatonin is administered, high concentrations are reached in the cell membrane, in the nucleus and in the mitochondria. Due to its indole chemical structure and high redox potential, melatonin can neutralise free radicals, thereby reducing oxidative stress and decreasing sepsis toxicity (Reiter et al., 2009).

Melatonin secreted in the pineal gland acts in the regulation of reproductive seasonality (Guerrero et al., 2007), having stimulatory effects in rams and male goats, which show reproductive activity in the autumn (Tsantarliotou et al., 2008; Reiter et al., 2009). The nocturnal secretion of melatonin in response to changes in the photoperiod is the passive message for the hypothalamic-pituitary-gonadal axis to identify which period of the year it is in (Harman and Serpek, 2022). The photoperiod is the common signal that determines the change of seasons in mammals from higher latitudes to the mid-tropics (Bronson, 2009). To date, melatonin receptors have been identified in neurons of the hypothalamus, in the pituitary gland, and in the testes and accessory sex glands (Gonzalez-Arto et al., 2017; Abecia et al., 2020; Yu et al., 2018). In the hypothalamus, where reproductive activity is modulated, the density of melatonin receptors may determine the sensitivity to changes in the photoperiod. Thus, species with numerous melatonin receptors in the central nervous system may use this neurohormone as a cue to circadian and photoperiodic rhythms, whereas species with lower numbers of receptors may use other cues to activate reproduction (Witt-Enderby et al., 2003).

To date, research on the benefits of using melatonin, either in the form of implants (Pool et al., 2020a, b) or added to chilled or frozen semen samples, has focused on reducing the oxidative stress induced by an excess of reactive oxygen species (ROS) in the seminal fluid (Ma et al., 2021; Casao et al., 2010a These ROS are generated mainly as a by-product of chemical reactions of spermatogenic processes, heat stress and the reproductive season (Casao et al., 2010b; Ofosu et al., 2021); such reactions cause oxidative damage to the germ cells, which is manifested by a decrease in blood flow, high levels of ROS and, ultimately, a decrease in semen quality (Pool et al., 2021; Shahat et al., 2022b). It is worth noting that melatonin is a ubiquitous molecule and found in the seminal plasma of rams, especially during the day, suggesting that it is secreted extrapineally by the reproductive tract (Gonzalez-Arto et al., 2016), considering that MT1 and MT2 receptors have been found mainly in the testis and seminal vesicle (Casao et al., 2010a). In this sense, when melatonin is used in the extender to chill and freeze semen samples in rams, sperm capacitation (Casao et al., 2010b) occurs with the addition of 1 µM melatonin, which causes a decrease in protein tyrosine phosphorylation and results in low levels of ROS and AMPc (Gimeno-Martos et al., 2019). However, in a more recent study by Pool et al., 2021, evaluating different levels of melatonin in ram semen, melatonin neither reduced the intracellular ROS levels after semen thawing nor lipid peroxidation, but it did reduce mitochondrial superoxide production and improved the motility and DNA integrity of frozen and thawed sperm (Medrano et al., 2017). In another study, 18-mg melatonin implants were able to increase the activity of the enzyme acrosin, which is important for initiating the acrosome reaction in the fertilisation process (Kokolis et al., 2000).

A second melatonin application is in the form of implants impregnated with melatonin, the use of which has been intensified both in sheep (Quintero et al., 2001; Ungerfeld, 2016) and rams (Pool et al., 2021) (Table 4). As sheep are a seasonal polyoestrous species, their reproductive activity is regulated by the photoperiod (Orihuela Trujillo, 2012; Leyva-Corona et al., 2023), and information about the photoperiod is transmitted to the gonadal system through the secretion of melatonin by the pineal gland. In rams, such implants can contain 18 mg of melatonin, and in one study with an implantation period of 14 weeks, the T levels increased until the 6^th^ week post-implantation, also affecting testicular size and the number of sperm per ejaculate, but not sperm motility and morphology (Pool et al., 2020b).

**Table 4:** Summary of the studies on melatonin, including breeds, doses, method of administration, time of the study and the main effects.

**Reference**	**Breeding season**	**Breed**	**Application method**	**Collection method**	**Collection time after application**	**Concentration**	**Main effects**
Palacín et al. (2008)	Outside	8 Soay	Subcutaneous implant	Seminal vesicles were removed	88 days	Silastic sheet (500-1DOw Corning sheeting, Midland, MI, USA) 1 g	After melatonin administration, emphasis was on the secretory epithelium characteristics. Induced a significant increase in the number and height of principal cells that showed signs of increased secretory activity. Main cytological alterations in the principal cells: increase in the concentrically arranged membranes of secretory vacuoles and glycogen granules, appearance of numerous lysosomes and multivesicular bodies
Casao et al. (2010a)	Outside	2 Aragonesa	Subcutaneous implant	Artificial vagina	66 to day 126	3, 18-mg melatonin implants (Melovine; Ceva Sanidad Animal, Barcelona, Spain)	Increased the percentage of progressive motile spermatozoa
	Outside	16 Dohne Merino	Subcutaneous implant	Artificial vagina	30 to day 78	3, 18-mg melatonin implants (Melovine; Ceva Sanidad Animal, Barcelona, Spain)	Increase in the scrotal circumference without changing the seminal quality parameters of fresh and frozen/thawed semen
Rekik et al. (2015)	Outside	7 Barbarine 10 Saint Croix rams	Subcutaneous implant	Artificial vagina	24 days	3, 18-mg melatonin implants (Melovine; Ceva Sanidad Animal, Barcelona, Spain)	Increased sperm concentration at the end of the experimental period. Scrotal circumference increased, and mating behaviour improved
Abecia et al. (2018)	Outside	6 Aragonesa	Subcutaneous implant	Blood	0-30 days	3, 18-mg melatonin implants (Melovine; Ceva Sanidad Animal, Barcelona, Spain)	Treated rams exhibited certain sexual behaviours and had higher plasma testosterone concentrations
							More of the ewes that mated with those rams became pregnant and produced more lambs per ewe compared to those that mated with non-treated rams
Pool et al. (2020a)	Outside	31, Merino and Poll Dorset	Subcutaneous implant	Blood	0 to day 72	Unmentioned	Increased blood plasma testosterone levels. Growth in testicular size and sperm count per ejaculation between weeks 3 and 12 after implantation. Reduced sperm DNA fragmentation in Poll Dorset rams during multiple weeks of the non-reproductive season
Abd-Elhafeez et al. (2021)	Outside	8 Soay	Sub- cutaneous implant	Seminal vesicles were removed	66 days	Silastic sheet (500-1DOw Corning sheeting, Midland, MI, USA) 1 g	Melatonin administration stimulated dendritic cells and macrophages through increasing the size and number of the endosomal compartments, which may correlate to increased immunity
Assani et al. (2023)	Outside	6 Touabire x Ladoum and Touabire x Peul Peul	Sub- cutaneous implant	Electroejaculator	60 to day 135	3, 18-mg melatonin implants (Melovine; Ceva Sanidad Animal, Barcelona, Spain)	Induced improvements in ejaculate volume, colour and appearance, sperm production and morphological abnormality rate; sperm motility and survival rate of spermatozoa were significantly improved

However, in a recent study using the same type of melatonin implant in both females and males, the group that had received the implant had significantly lower values for first oestrus, conception rate and lambing compared to the control animals (Tölü et al., 2022).

In another study carried out by Abecia et al., 2018, in rams, where a light programme was combined with melatonin implants, the rams that had received the light treatment plus the placement of three melatonin implants still presented the highest levels of plasma T at 15 and 30 days after exposure to the natural photoperiod; the ewes introduced to the melatonin-treated rams had a lambing percentage of 100% and a high fertility (1.44 ± 0.51 lambs/ewe) compared to the control ewes (78% lambing and 1.00 ± 0.69 lambs/ewe). In the same direction, but in young lambs, the use of implants with 18 mg of melatonin does not improve the sexual behaviour of rams, but when exposed to a batch of ewes, the pregnancy rate was 89%; in addition, treated lambs had better testicular development and higher T concentrations, indicating that the use of melatonin implants in lambs can reduce the variability of age at sexual maturity [98]. However, more recent studies have evaluated different doses of melatonin (18, 36 and 54 mg) in adult Border Leicester rams, and rams receiving 36- and 54-mg melatonin implants showed an increase in scrotal circumference and T concentration, indicating the need to use higher doses of melatonin and that the 36-mg dose is required to provoke an adequate response of testicular size (Kleemann et al., 2021; Kleemann et al., 2022). In another study, melatonin implants (36 mg) increased testicular volume, which is important in performing testicular blood perfusion (TBP) studies for the diagnosis of reproductive disorders and in assessing ram fertility (Akar et al., 2023). Table 4 shows a summary of some studies focussing on the use of melatonin in the form of implants, summarising its positive effects on semen quality and T concentration.

## Conclusions and future implications

With the increasing demand for animal feed, the use of hormonal technologies to make ram reproduction more efficient has accelerated in recent years. This implies that the hormonal products mentioned in this review should always be accompanied by optimal animal welfare measures and should not affect meat quality. For all hormonal products, there is a wide variation in the length of time each has been evaluated and the impact it has on ram reproduction. The reason for presenting six of the most important hormones used to improve ram performance was to establish the current state of research on the effects of each of these hormones on ram reproduction and to define their use in the immediate future, with a view to achieving a balance between animal welfare and flock productivity on a global scale.
